# Advancements in application of modified biochar as a green and low-cost adsorbent for wastewater remediation from organic dyes

**DOI:** 10.1098/rsos.232033

**Published:** 2024-05-15

**Authors:** Kosar Hikmat Hama Aziz, Nazhad Majeed Fatah, Khalid Taib Muhammad

**Affiliations:** ^1^ Department of Chemistry, College of Science, University of Sulaimani, Qlyasan Street, Sulaymaniyah City, Kurdistan Region 46001, Iraq; ^2^ Medical Laboratory Analysis Department, College of Health Sciences, Cihan University-Sulaimaniya, Sulaymaniyah, Kurdistan Region 46001, Iraq; ^3^ Department of Environmental Science, College of Environmental Sciences, University of Sulaimani, Sulaymaniyah-Chwarta 46001, Iraq; ^4^ Department of Natural Resources, College of Agricultural Engineering Sciences, University of Sulaimani, Sulaymaniyah 46001, Iraq

**Keywords:** adsorption, biochar, modified strategy, wastewater remediation, organic dyes

## Abstract

Synthetic organic dyes, which are resistant to biodegradation, pose a notable health risk, potentially leading to cancer and respiratory infections. Researchers have addressed this concern by exploring physicochemical methods to remove organic dyes from wastewater. A particularly promising solution involves modified biochar adsorbents, which demonstrate high efficiency in organic dye removal. Biochar, a charcoal-like material derived from biomass pyrolysis, offers advantages such as low cost, eco-friendliness, high efficiency and reusability. Beyond its role in sustainable soil remediation, biochar proves effective in removing organic dyes from wastewater after undergoing physical or chemical modification. Acid–base activation or metal–heteroatom impregnation enhances biochar's adsorption capacity. This comprehensive review examines the attributes of biochar, common methods for production and modification, and the impacts of raw materials, pyrolysis temperature, heating rate and residence time. It further elucidates the biochar adsorption mechanism in the removal of organic dyes, assessing factors influencing efficiency, including biochar feedstock, solution pH, adsorption temperature, particle size, initial dye concentration, biochar dosage and reaction time. It explores challenges, opportunities, reusability and regeneration methods of biochar in treating organic dye wastewater. It also discusses recent advances in organic dye removal using adsorption-based biochar. The review ultimately advocates for enhancing biochar's adsorption performance through post-modification.

## 1. Introduction

Water pollution is a major problem that is exacerbated by rapid economic growth and population growth. The use of fossil fuels in many industries, as well as the expansion of agriculture and manufacturing, produces a variety of pollutants that can harm human health and the environment [1,2]. Water pollution can be caused by industrial activities, which release untreated or partially treated wastewater into the environment. This wastewater can contain harmful pollutants, such as toxic inorganic and organic compounds [3,4]. Synthetic dyes, exceeding 100 000 in global availability, are extensively employed across various industries such as textiles, tanning, printing and paper [5]. They also play roles in pharmaceuticals, cosmetics and food processing, with some dyes having medicinal applications. Water pollution can stem from various sources, including industrial, agricultural, domestic and clinical effluents. Industrial effluents, especially in rapidly urbanizing, globalizing and densely populated regions, emerge as major contributors to water contamination [6]. Concern arises from the diverse pollutants produced by industrial activities, including harmful synthetic organic dyes that pose risks to human health and the environment [7,8]. Textile wastewater often contains abundant synthetic dyes, making it a significant source of industrial organic dye wastewater [9]. Different types of organic dyes can be categorized based on their chromophores, such as azo dyes, anthraquinone dyes, nitro dyes and phthalocyanine dyes, among others. These categories exhibit unique chemical structures and characteristics that impact their colour and performance across different uses [10]. Organic dyes, not always securely bound to fabrics, can be released into water during rinsing in textile processes. As a result, wastewater from textile industries frequently contains high concentrations of diverse dyes [11,12]. This pollution can adversely affect water resources, posing environmental challenges and harming the aquatic environment due to the potential toxicity and persistence of these dyes [13]. Most organic dyes are harmful as they resist biological degradation and can accumulate in the body, leading to health issues such as cancer and respiratory infections. The persistence and poor degradability of reactive organic dyes present a significant environmental threat by bypassing conventional wastewater treatment plants and entering ecosystems [14]. The inherent toxicity, associated with adverse effects like mutagenesis, chromosomal damage, carcinogenicity and respiratory problems, underscores the pressing need for effective strategies to remove dyes from wastewater streams [15,16]. Efforts should be focused on developing effective treatment methods to reduce the environmental impact of dye-containing wastewater. Researchers should favour low-cost and sustainable technologies to efficiently mitigate the environmental risks posed by organic dye pollutants at the source before discharging them into aquatic ecosystems. Various treatment methods have been developed and used to remove organic pollutants from aqueous solutions, including physical, chemical, biological and hybrid approaches. Several review articles have discussed the removal of synthetic organic dyes from wastewater using physical adsorption [17–20], biological degradation [10,21] and advanced chemical oxidation processes [22,23], such as photocatalysis [24,25], ozonation [26], sulfate-radical [27,28] and Fenton and Fenton-like oxidation [29,30]. Table 1 summarizes the advantages and limitations of various treatment methods for organic dye wastewater treatment.

**Table 1.** Advantages and limitations of various treatment approaches for water remediation using organic dyes.

methods	influencing conditions	advantages	limitations	ref.
advanced oxidation processes	design of the reactor, solution pH, initial concentration, target pollutant, and power discharge in non-thermal plasma	efficient decolorization and mineralization with no secondary pollution	high energy consumption is prone to maintenance, generates sludge, and is expensive for industrial-scale implementation	[31]
ozonation	levels of O_3_, solution acidity, rate of gas flow, amount of catalyst, and composition of the catalyst	application of O_3_ gas under ambient conditions and scalability	expensive, limited mineralization, selective degradation, dependent on pH	[32]
membrane filtration	membrane type, organic dye type, and constituents of wastewater	provides easy operation, versatility, scalability, operates at ambient temperature, and seamlessly integrates with current treatment systems	significant energy requirements, generation of concentrated sludge, and fouling of the membrane	[33]
coagulation/flocculation	necessitates the use of chemical coagulants	economical, easily scalable, simple and accessible, and suitable for a broad spectrum of dyes	issues with sludge formation, secondary treatment, susceptibility to pH and ionic strength, and occasional lack of environmental friendliness	[34]
extraction	composition of the solvent, pH levels, temperature, ionic strength, and the type of organic dye	selective, high efficiency	incomplete elimination, the potential for secondary pollution, elevated expenses, and a lack of scalability	[35]
biological degradation	microbiological degradation-based treatment	environmentally friendly and low cost	process is slow, demanding ample nutrients, constrained by a narrow temperature range, and requiring a significant land area	[36]
adsorption	amount of adsorbent used, the initial dye concentration, pH, temperature, and the water matrix	excellent removal of a wide variety of dyes	regeneration is necessary, and the disposal of the adsorbent is expensive	[37]

**Figure 1.**
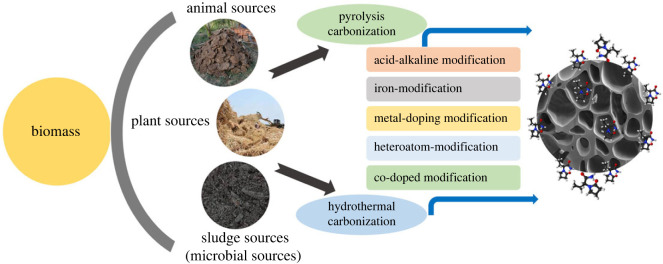
Biochar production and modification from various feedstocks for organic dye adsorption.

The treatment methods outlined in table 1 demonstrate effectiveness in removing organic dyes from wastewater. Each approach has its own set of advantages and disadvantages in practical application. Among the mentioned treatment methods, adsorption utilizing green adsorbent catalysts emerges as the most favourable approach. This is attributed to its simplicity, cost-effectiveness, low energy requirements, recyclability, waste treatment with a waste strategy, resource utilization, and versatility in addressing a wide range of pollutants [12]. This technique has proven particularly effective in treating organic dye wastewater. Biochar, a carbon-rich pyrogenic material produced from biomass in a low-oxygen environment, has recently gained significant attention due to its diverse applications and advantages in agriculture, environmental remediation, and carbon sequestration [38,39]. Biochar is characterized by its elevated specific surface area, well-developed pore structure, and abundant functional groups such as hydroxyl, carboxyl, and phenolic groups, which impart excellent adsorption properties for organic and inorganic pollutants in soil and aqueous systems [40]. Biological wastewater treatment methods face challenges in effectively removing most organic dye residues due to their recalcitrant nature and biological degradation. This limitation underscores the imperative for developing sustainable and efficient technologies capable of eliminating complex organic dye pollutants. This review highlights the biochar-based adsorption process as a promising solution, offering efficiency, cost-effectiveness and environmental friendliness in the removal of organic dyes from aqueous environments [40–43]. The review focuses on recent research exploring the production, modification, characterization and application of biochar for the remediation of water contaminated with organic dyes (figure 1).

**Figure 2.**
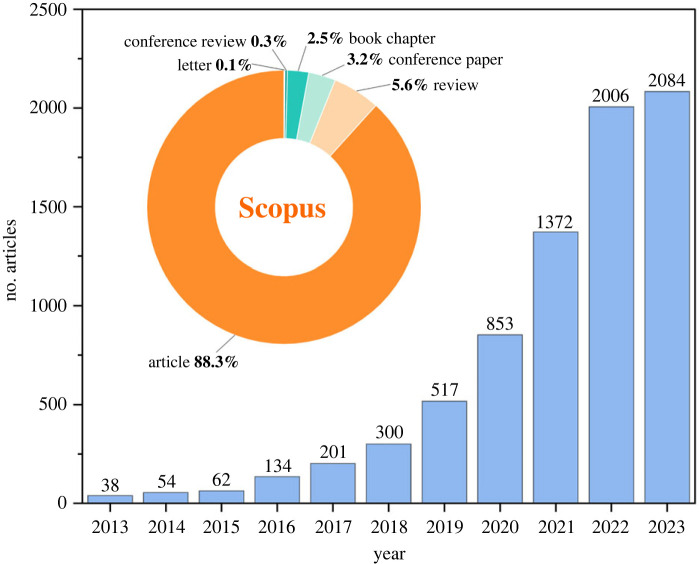
The articles published in recent 10 years with the topic of biochar and adsorption and dyes in 2013–2023. Source: Science Direct and Scopus.

The biochar-based adsorption method is gaining attention for its capability to remove various pollutants, particularly organic dyes [44]. Figure 2 illustrates the continuous increase in publications on biochar-related research focusing on the adsorptive removal of organic dyes from 2013 to 2023. These trends suggest a rising interest in utilizing biochar for removing organic dyes from wastewater, attributed to its advantages such as high efficiency and cost-effectiveness in adsorption methods using biochar-based adsorbents. The overall trajectory indicates significant advancements in biochar-based adsorbent research for removing organic dyes. Analysis of frequently cited literature reveals that key research areas in the past decade have included water contamination remediation, biochar structure and properties, as well as adsorption performance and reactivity [45]. The availability of feedstocks like agro-industrial wastes enables the production of eco-friendly biochar. The proven effectiveness of both biochar and modified biochar in environmental remediation highlights their potential for removing organic dye pollutants from water sources.

This review summarizes recent applications of biochar and modified biochar in treating organic dye wastewater. The review delves into the use of modified biochar adsorbents and provides insights into post-synthesis modification strategies, enhancing surface area, improving porosity, and surface functional groups in biochar adsorbents for improved environmental friendliness. Additionally, the paper explores common mechanisms in biochar-based adsorption processes for treating organic wastewater and suggests strategies for regenerating and stabilizing modified biochar. It identifies limitations, proposes future directions for advancements, and underscores the ongoing exploration of modified biochar to address water pollution from organic dyes. The review specifically highlights prospective research on applying biochar materials for removing organic dyes from industrial wastewater.

## 2. Organic dye pollution in aquatic environments

**Figure 3.**
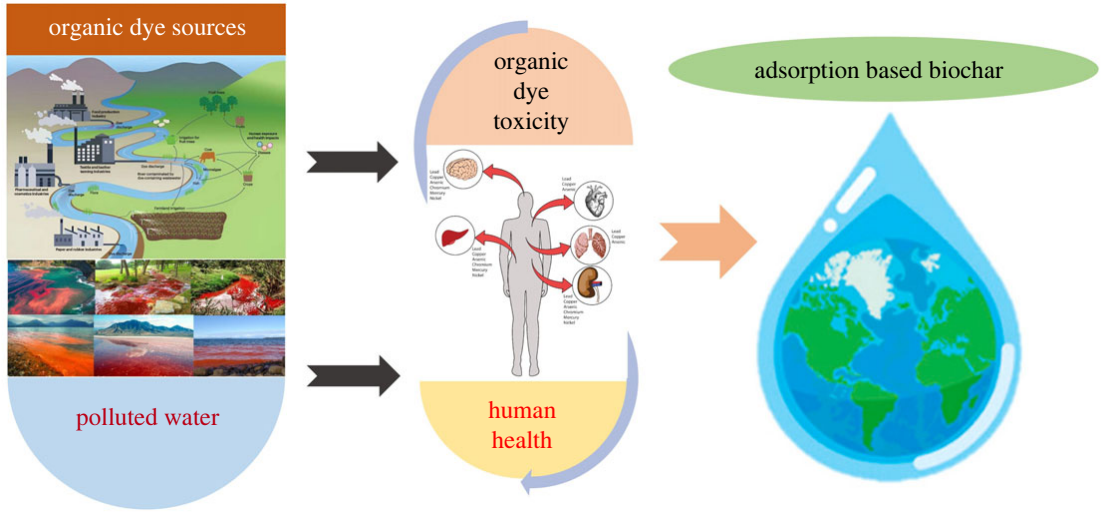
Origin, oxicity, and adsorption-based biochar treatment of organic dyes in water.

The extensive global production and use of synthetic organic dyes, along with their broad applications, lead to their presence in aquatic environments. Dye compounds have various sources, both direct and indirect, in the water environment (figure 3). Key direct sources include textile dyeing, tannery, printing, cosmetics, personal care products, and paper industries, which discharge substantial volumes of wastewater, contributing to water contamination in aquatic ecosystems [46,47]. Household wastewater contributes synthetic organic dyes to aquatic environments through the disposal of expired or unused dye-derived drugs, hair dyeing, and the use of cosmetics or household chemicals containing dyes. These substances are discharged into sewers, eventually reaching sewage treatment plants or being directly released into the environment [48,49]. In various dyeing processes, the wastage of dyes ranges from a minimum of 5% to as much as 50%, depending on factors like fabric type and dye characteristics. Consequently, the industry produces nearly 200 billion litres of coloured effluent annually [48]. Organic synthetic dyes and pigments are also used in the plastic, leather, ceramic, cosmetic, and food processing industries. However, a significant amount of these dyes are discharged into the environment during synthesis and industrial processing, resulting in serious environmental problems worldwide [50,51]. More than 100 000 dyes are in commercial circulation, with an annual production exceeding 700 000 tons. Textile and dyeing processing factories alone account for over 60% of dye consumption, driven by rapid industrial and urban expansion. Approximately 20% of these dyes are released into water bodies as effluents during manufacturing and application processes, as indicated by a recent study [52]. The characteristics of organic dye wastewater vary widely based on the equipment used, the nature of raw materials, and the processes of production, compounding, and formulation. In addition to dye residues, wastewater from textile manufacturing industries frequently contains significant amounts of hazardous organic and inorganic chemicals, comprising both biodegradable and non-biodegradable components [9,53]. Organic dyes can harm aquatic life by reducing sunlight penetration into the water and inhibiting photosynthesis. Additionally, most organic dyes are chemically stable, difficult to biodegrade, and toxic to the environment due to their resistance to aerobic degradation and the formation of carcinogenic aromatic amines during anaerobic degradation [54,55]. Currently, there is limited information available regarding the presence of synthetic organic dyes in aquatic environments. Dyes have been identified in various water samples, including rivers, drinking water, and wastewater, as well as in other samples such as sediments, soils, and wild fish. Previous studies suggest that some organic dyes exhibit toxic properties, including carcinogenic, allergenic, and dermatological effects, raising safety concerns associated with the production and use of these compounds [56,57]. During degradation processes, synthetic organic dyes undergo varied patterns of transformation kinetics, with some producing more toxic degradation products than the original compounds (e.g. vat green 3). Conversely, others may initially transform into more toxic forms but subsequently convert into non-toxic compounds (e.g. food red 17) or directly metabolize into non-toxic products (e.g. food yellow 3) [58]. Textile dyes frequently comprise a combination of dyes derived from diverse chemical classes, thereby suggesting that their influence on aquatic organisms might vary when contrasted with that of individual organic dyes [59]. Conducting a comprehensive risk assessment of synthetic organic dyes, with a particular focus on their impact on aquatic life, is of utmost importance. This is critical because dyes present in water ecosystems have the potential to jeopardize the health of animals or humans as they move up the food chain. To assess the influence of synthetic organic dyes on water bodies, it is crucial to consider the toxic effects of these dyes on aquatic organisms at various trophic levels. Environmental protection and safety guidelines from a reputable global organization recommend testing the aquatic toxicity of organic dyes on algae, plants, invertebrates and vertebrates to evaluate environmental hazards in aquatic environments [48]. The existence of synthetic organic dyes in aquatic environments poses a significant environmental challenge. Our knowledge about the impact of synthetic organic dyes on water organisms and plants remains incomplete. Given the extensive variety of synthetic organic dyes, current ecotoxicological studies only address a fraction of the total dyes in use. The substantial water consumption in industries and the loss of dyes during textile processes result in substantial volumes of coloured effluents that require treatment before being released into the aquatic environment. Figure 3 provides an overview of sources, toxicity and the utilization of biochar for adsorption in the treatment of organic dyes in wastewater.

## 3. Various adsorbents in the removal of organic dyes from water

Developing efficient and sustainable methods for removing organic dyes is crucial. Various techniques exist for their recovery or destruction, encompassing chemical, physical, and biological approaches [60,61]. Numerous wastewater treatment technologies for organic dye removal, including adsorption [62,63], membrane technology [64], advanced oxidation processes [14] and biodegradation [65], have been developed. However, the complexity and diversity of dyes make finding a single, universally effective solution a significant challenge. Organic dye reduction methods can be categorized into destructive and recovery approaches. Recovery methods mainly involve physical treatments utilizing techniques like adsorption, membrane separation, and absorption. On the other hand, destructive methods encompass chemical processes such as advanced catalytic oxidation and non-thermal plasma oxidation, as well as biological methods. Chemical oxidation and biological degradation aim to transform organic dyes into less harmful compounds or easily manageable byproducts. However, the industrial application of chemical methods faces challenges such as high energy consumption and the generation of undesired byproducts. In some instances, the oxidation byproduct may be more toxic and harmful than the initial dye pollutants [8,66,67]. Therefore, the use of adsorption processes for water remediation from organic dyes is considered the most suitable due to their simple design, cost-effectiveness, low energy consumption, and broad processing range. Such adsorption processes have found extensive use in controlling various organic pollutants in water systems. Table 2 illustrates various adsorbents employed in the literature for the treatment of diverse organic dyes in aqueous solutions. The results from published studies clearly indicate that the specific surface area significantly influences the adsorption capacity of the adsorbents. Other factors, such as porosity, pore size, and pore distribution, as well as the raw materials used in the preparation of the adsorbent, affect the types and number of functional groups on the adsorbent surface. These factors are incorporated into the interaction forces between the adsorbent and dye pollutants, significantly affecting their adsorption performances.

**Table 2.** Various adsorbents used in removal of organic dyes from aqueous solution.

adsorbent	surface area (m^2^ g^−1^)	reaction condition	organic dye	capacity (mg g^−1^)	ref.
metal–organic framework (MOF)	166.6	Zr-MOFs, pH = 7, [100] mg l^−1^	malachite green	234.2	[68]
CoCr_2_O_4_ nanoparticles	8–17 nm	8 nm catalyst, pH = 7, [30]_o_ mg l^−1^	Congo red	59.4	[69]
ZIF-8 MOF/PVA composite	1704.03	0.4 mg ml^−1^, pH = 3–10, [50]_o_ mg l^−1^	Congo red	829.39	[70]
natural Iraqi bentonite clay	30.6	pH = 5.6, [150] mg l^−1^	methylene blue	253.2	[71]
chitosan/coal fly ash composite	2.12	adsorbent dose: 0.07 g at 45°C, pH = 4, [100] mg l^−1^	reactive red 120	237.7	[72]
Chinese yam peel-modified polypyrrole	79.96	adsorbent dose: 10 g l^−1^, at 45°C, pH = 4–7, [100] mg l^−1^	Congo red	86.66	[73]
breadfruit peel	—	adsorbent dose: 0.04 g at 25°C, pH = 11, [250] mg l^−1^	crystal violet	176.25	[74]
agricultural waste biomass (corn leaves, groundnut shells, and coconut coir)	484.14, 93.45, and 62.14	adsorbent dose 5 g l^−1^, at 25°C, pH = 6.5, [100]_o_ mg l^−1^	acid blue dye 113	101–150	[75]
perovskite	66.67	LaCoO_3_ perovskite loaded onto g-C_3_N_4_ at 25°C, pH = 7, [100] mg l^−1^	rhodamine blue	1226	[76]

The selection of adsorbents is primarily determined by their low cost and environmental friendliness. Traditional materials like activated carbon, silica gel, modified zeolite, alumina, polymer resin and others have been widely used for water treatment through adsorption [77]. However, their application faces challenges such as high cost, low selectivity, complex regeneration and limited reuse performance. Carbonaceous materials like activated carbon, carbon nanotubes, graphene and biochar have gained prominence as a subject of research in the adsorption field, owing to their non-toxicity and the lack of secondary pollution during treatment [78]. Commercial activated carbon, a common choice for decades, faces challenges due to its high cost. While scientists have created different sorbent materials, only a few have become practical technologies, limited by technical constraints and variations in real-world applications [79,80]. Biochar, emerging as a low-cost pollutant adsorbent, is gaining prominence. Recent studies show that laboratory-produced biochar can adsorb organic dye pollutants comparable to commercial activated carbon [81,82]. Biochar and its modified composites present a new, cost-effective and environmentally friendly wastewater treatment technology.

## 4. Biochar production

Over the years, activated carbon has been widely used in adsorption processes. However, its expensive production and the environmental impact of coal transportation to end users are concerning [83]. Biochar, an eco-friendly alternative to activated carbon, offers abundant availability, low cost, high porosity, multiple functional groups and excellent resistance to acid and alkali corrosion, making it a promising adsorbent [84,85]. Mesoporous and microporous structures are crucial features of biochar, impacting its surface area and functionality [86]. These pores increase biochar's surface area, enabling better pollutant interaction and enhancing adsorption capacity, particularly in environmental remediation. The choice of feedstock biomass and pyrolysis conditions can alter biochar properties, such as surface area and pore distribution (micropores, <2 mm; mesopores, 2–50 nm; macropores, >50 nm), thereby influencing its potential applications. Macropores facilitate substance diffusion, mesopores serve as mass transfer channels and micropores offer trapping space [87]. The physical characteristics of biochar, including its specific surface area and porosity, are important factors for various applications like wastewater treatment. There is considerable focus on producing engineered biochar with heightened porosity and extensive specific surface area. Engineered biochar holds significant promise for a range of environmental applications, particularly in wastewater treatment [88]. Produced using simple biomass pyrolysis methods, biochar showcases unique characteristics like its porous structure and sufficient surface area, rendering it highly efficient in adsorbing and removing organic dyes from wastewater [42]. The properties and production rates of biochar are significantly influenced by the attributes of the initial biomass wastes and the operational parameters of the pyrolysis process, including temperature, heating rate and residence time [89]. Biochar typically exhibits surface areas and pore volumes ranging from 8 to 132 m^2^ g^−1^ and 0.016 to 0.083 cm^3^ g^−1^, respectively. However, with appropriate biomass feedstock and optimal pyrolysis conditions, biochar can achieve surface areas of up to 490.8 m^2^ g^−1^ and total pore volumes of 0.25 cm^3^ g^−1^ [86]. Post-treatments like KOH activation significantly enhance biochar engineering, increasing surface area and total pore volume to 3263 m^2^ g^−1^ and 1.772 cm^3^ g^−1^, respectively, as demonstrated in a study by Liu *et al.* [90], comparable to or exceeding those of commercial activated carbon. Biochar pore volume comprises micropores (0.012–0.060 cm^3^ g^−1^) and mesopores (0.007–0.020 cm^3^ g^−1^), with respective median values of 0.024 cm^3^ g^−1^ and 0.009 cm^3^ g^−1^ [86]. Effect of operating conditions on biochar production during pyrolysis includes the following.

### 4.1. Temperature

The temperature during pyrolysis significantly influences the chemical compositions and surface characteristics of biochar. It has been established that the presence of various functional groups, such as hydroxyl, carbonyl, amide and amines, on the biochar surface plays a crucial role in removing pollutants from wastewater. At elevated pyrolysis temperatures (e.g. ≥800°C), the oxygen-containing functional groups on biochar are effectively diminished, resulting in the formation of aromatic structures with higher degrees of carbonization. Consequently, biochar derived from high temperatures exhibits heightened hydrophobicity and aromaticity, often showcasing exceptional adsorption capabilities for organic contaminants [91]. However, an elevated temperature may induce chemical rearrangement in biochar. This restructuring can result in the breakdown of the biochar structure, blocking its pores and subsequently diminishing its adsorption capabilities [92]. In contrast, biochar produced at a comparatively low pyrolysis temperature (e.g. ≤450°C) possesses a greater abundance of surface functional groups compared to biochar synthesized at higher temperatures. These functional groups offer numerous active sites for the adsorption of pollutants from wastewater [93]. The heating rate affects biochar yield during pyrolysis, with higher rates leading to decreased biochar production. This decrease is offset by increased volatile yield. The shift is attributed to chemical bond stability, as faster heating rates break biopolymer bonds in biomass more rapidly, reducing biochar yields. Conversely, at lower heating rates, stronger bonds remain intact, inhibiting volatile formation and increasing biochar yield [94]. Solar *et al*. noted that at low pyrolysis temperatures, the increase in residence time has minimal impact on char yield. However, longer gas residence times can raise bio-oil yield by prolonging pyrolysis duration, consequently reducing biochar yield. Conversely, extended vapour residence times may induce secondary tar cracking, resulting in decreased bio-oil yield, while promoting polycondensation reactions to produce more char, thereby increasing biochar yield [95].

### 4.2. Biomass feedstocks

Biomass feedstocks for biochar production include agricultural residues, animal manure, forestry residues, sludge, wastepaper and diverse industrial wastes, mixing organic and inorganic compounds [96]. These resources provide a sustainable and renewable source for biochar production. Woody biomass consists of forestry residues and tree materials, while non-woody biomass comprises crop residues, other agricultural and animal wastes, and industrial and urban solid wastes [97]. Woody biomass mainly contains lignin, cellulose and hemicellulose, along with varying minerals and other elements affecting biochar production. Woody biomass generally has high bulk density and low moisture and ash content, whereas non-woody biomass tends to have high moisture and ash levels and low bulk density [39]. The type and composition of biomass feedstock have a considerable impact on both the properties and yield of biochar. Research suggests that differences in feedstocks and pyrolysis conditions play a significant role in shaping the qualities and quantities of the resulting biochar [98]. Thermochemical processes can transform lignocellulosic biomass into biochar. This biomass primarily consists of carbohydrate polymers like cellulose and hemicellulose, aromatic polymers such as lignin, extractives like resins, tannins and fatty acids, alongside inorganic elements. Biomass with higher lignin content generally yields more biochar [96]. Biochar derived from plant sources, particularly lignin-rich biomass like bamboo and coconut shells, tends to exhibit larger pore sizes. Conversely, biochar obtained from cellulose-rich biomass such as rice husks typically features smaller pores [99]. Biochar obtained from sludge typically contains more ash than that from plant and animal sources, likely due to the intricate composition of sewage sludge. Sludge-derived biochar commonly includes nitrogen doping and metal loading, particularly Fe and Al, originating from prevalent flocculants like polyacrylamide, polymeric ferric sulfate and polyaluminium chloride used in sludge chemical conditioning [100]. Biochar derived from animal manures demonstrates elevated concentrations of elements such as phosphorus, magnesium, calcium and potassium when compared to biochar from plants [101].

## 5. Biochar modification techniques

Typically, biochar produced directly through biomass pyrolysis exhibits limited surface functionality, low porosity and a small surface area, severely restricting its applications in environmental remediation [40]. Extensive research has focused on surface modification and functionalization of biochar to overcome this limitation. Unmodified biochar often has limited capacity and efficiency in eliminating organic pollutants. Treatment with chemicals or other materials enhances the adsorption capacity of modified biochar [102,103]. Considering the limitations of biochar concerning its adsorption performance, different modification techniques are employed to produce biochar composite materials that exhibit enhanced adsorption capabilities, leading to increased economic advantages in engineering applications. Modifying biochar is a crucial procedure aimed at enhancing its adsorption capacity, activation potential and both physical and chemical properties. The methods employed for modification exert significant effects on the physicochemical attributes of biochar [104]. This alteration process not only impacts the surface functional groups and specific surface area of biochar but also alters the pore structure and size distribution. Table 3 illustrates the effect of modification techniques on the adsorption capacities of biochar for removing various organic pollutants from water.

**Table 3.** Adsorptive removal of organic dye pollutants from water using untreated and modified biochar.

[organic dye]_o_	persistent biochar	capacity (mg g^−1^)	modified biochar	capacity (mg g^−1^)	ref.
methylene blue (50 mg l^−1^)	unmilled bagasse biochar	17.20	ball-milled bagasse biochar	354.00	[105]
methylene blue (100 mg l^−1^)	sorghum straw biochar	43.28	Fe_3_O_4_-loaded biochar	128.51	[106]
methylene blue (100 mg l^−1^)	chitosan-loaded biochar	8.83	KOH-activated biochar	62.04	[107]
Congo red (50 mg l^−1^)	*Acacia auriculiformis* biochar	4.00	FeCl_3_-modified biochar	130.00	[108]
Congo red (500 mg l^−1^)	Medulla Tetrapanacis biochar	580.83	urea/CaCl co-modified biochar	2512.82	[109]
Congo red (50 mg l^−1^)	green pea peel biochar	62.11	ZnO-green pea peel biochar	114.94	[110]

**Figure 4.**
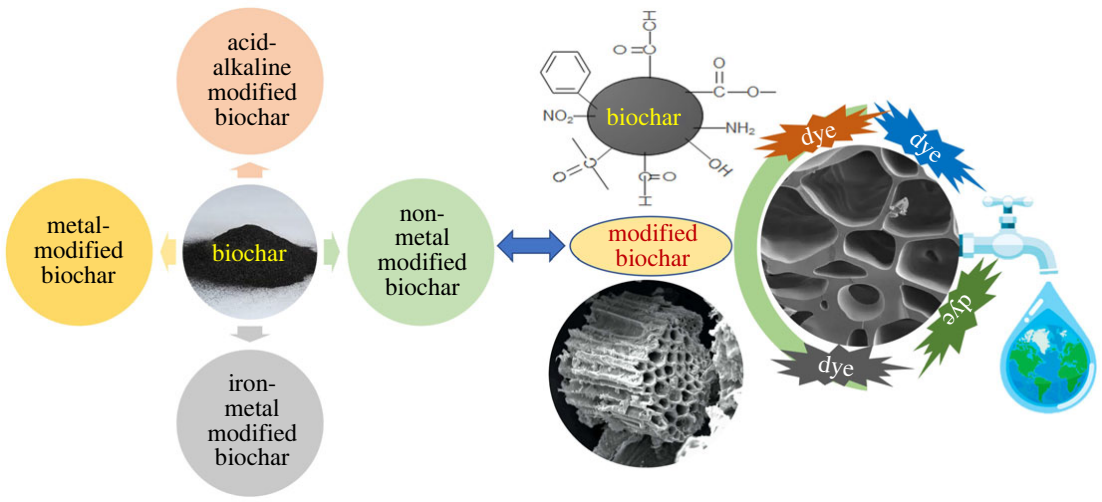
Various modification strategies aim to enhance the efficiency of biochar in adsorbing organic dyes from aqueous solutions.

Several modification techniques have been developed, directly influencing the adsorption capacity and mechanism of biochar for removing pollutants. Therefore, selecting a suitable biochar modification method is crucial, considering the physical and chemical properties of emerging contaminants in wastewater for efficient removal [111]. Commonly used modification methods include chemical modification such as iron-loaded magnetic adjustment, acid–alkaline modification, as well as metal and nonmetal modifications (figure 4) and physical modification methods such as steam, ball-milling and carbon dioxide activation. Following these modifications, the specific surface area, porosity and surface functional groups of biochar undergo improvements. Magnetic modification enables straightforward separation and boosts removal efficiency. Acid–base modification improves the active functional groups, pore structure and surface area of biochar adsorbent [112]. The modification of biochar through metal doping enhances the removal rates of organic dyes and imparts magnetism. This modification proves effective for multiple cycles, enabling the removal of both heavy metals and organic dyes from water and wastewater [113]. Heteroatom-doped biochar increases adsorption capacity by augmenting specific surface area and the number of functional groups. Grim *et al*. synthesized nitrogen-doped biochar utilizing shiitake spent mushroom feedstock. Raman spectroscopic examination indicated that the nitrogen-doped biochar exhibited increased defective carbon structures compared to the non-doped counterparts. X-ray photoelectron spectroscopic analysis revealed that the introduction of melamine during the doping process resulted in the formation of nitrogen functionalities on the biochar particle surface. This enhancement contributed to the effective removal of contaminants from both synthetic effluents and sewage water, demonstrating good recyclability [114]. Through the utilization of diverse modification techniques, a biochar catalyst can exhibit enhanced specific surface area and augmented functional groups to attain optimal adsorption efficiency. If the objective of the modification is to introduce additional nitrogen–carbon functional groups, employing nitrogen-rich raw materials, such as waste from animal manure, can be beneficial.

### 5.1. Acid–alkaline modified biochar

Chemical modification with acids or bases can enhance the adsorption capability of biochar. The feedstock may undergo treatment before or after pyrolysis to produce activated or modified biochar. Acid treatment of biochar involves immersing it in a solution of acid, typically at elevated temperatures up to 120°C. This process can be quite extensive, sometimes requiring several days of continuous contact between the acid and the biochar [41]. Acid treatment of biochar can influence its surface charge due to the introduction of additional functional groups. Despite being predominantly alkaline in nature, biochar possesses a naturally negatively charged surface. This negative charge becomes even more amplified when treated with acid, making the surface even more electronegative [115,116]. Alkaline activators can significantly improve the pore size, specific surface area and surface alkalinity of biochar catalysts. The alkali treatment enlarges micropores into mesopores by inducing carbon loss, creating a pathway for adsorbent transport, resulting in enhanced adsorption capacity [117,118]. Alkali modification has been employed in various stages of biochar production, serving distinct purposes. Following alkali pretreatment, the hydrophilicity of biochar diminishes, allowing it to adsorb organic pollutants through hydrophobic interactions. On the other hand, alkali post-treatment typically introduces oxygen-containing functional groups to biochar, contributing to the effective removal of both organic and inorganic pollutants [119–121]. Activated biochar exhibits improved surface area, enhanced chemical functionality and high-affinity adsorption sites, promoting effective interaction with pollutants [122]. Chemical agents like potassium hydroxide, phosphoric acid, or malic acid can be applied to the raw materials or introduced during pyrolysis to create modified biochar with increased adsorption efficiency for removing contaminants [123,124]. Researchers showed that subjecting the biochar, along with the KOH blend, to pyrolysis at temperatures ranging from 350 to 550°C reopened some of its blocked pores and widened the smaller pore sizes, resulting in increased surface area [125]. Tabassam *et al*. utilized rice husk-derived biochar modified with cinnamic acid. The introduction of carboxylic groups by the acid proved advantageous for the adsorption of cationic methylene blue dye from aqueous solutions. The findings indicated that the adsorption mechanism is grounded in ion exchange, hydrogen bonding, π–π interactions and electrostatic interactions. A maximum adsorption capacity of 277.8 mg g^−1^ was achieved. The study suggests that rice husk biochar modified with cinnamic acid holds promise as an effective adsorbent for the removal of methylene blue from aqueous mediums [126]. Zhang *et al*. conducted the synthesis of activated modified biochar from wood modified with phenol–formaldehyde resin through pyrolysis. The biochar prepared underwent activation using potassium hydroxide and was utilized for adsorbing Congo red (3472.22 mg g^−1^) and methylene blue (1112.35 mg g^−1^) dyes. Alkaline and acidic treatments enhance the presence of oxygen-loaded functional groups on the surface of biochar. This modified biochar exhibits an impressive porous structure with abundant oxygen-containing functional groups, leading to a significantly elevated specific surface area and total pore volume. Additionally, the modification process induces greater disorder and creates more defect sites in the activated wood biochar, which are essential for efficient adsorption during the treatment of organic dyes in wastewater [127].

### 5.2. Iron-modified biochar (magnetic biochar)

Biochar magnetization represents an exciting frontier in biochar modification. Biochar poses a challenge in terms of separation after wastewater treatment, primarily due to its small volume, which can lead to secondary pollution and significantly hinder its practical application. To address this issue, recent research has focused extensively on magnetic biochar. The incorporation of magnetic active materials, such as iron and manganese, into biochar through modification processes offers numerous advantages, including favourable physical and chemical properties, a large specific surface area, high pollutant affinity and easy separation [104,128]. This approach holds considerable promise for diverse applications in wastewater treatment. Magnetic biochar is an innovative bio-carbon material that possesses both favourable adsorbent properties and magnetic characteristics. The utilization of magnetic-modified biochar has been investigated to improve particle separation efficiency in wastewater post-treatment [46]. Magnetic biochar, a composite material incorporating magnetic elements like Fe_3_O_4_, encompasses the favourable characteristics of traditional biochar along with added magnetic properties [129,130]. Comprising two primary components, the biochar portion possesses a high surface area, well-developed porous structure and abundant surface functional groups, enhancing its effectiveness as an adsorbent. The magnetic component facilitates easy separation, addressing the challenge of separating conventional biochar from water post-treatment [131,132]. The incorporation of magnetic components has a notable impact on the physical and chemical characteristics of biochar. The synergistic qualities of biochar and magnetic materials render magnetic biochar suitable for the removal of pollutants, including organic dyes, from wastewater. Numerous studies have consistently demonstrated the effectiveness of magnetic biochar as a proficient adsorbent, showcasing outstanding performance in the adsorption of organic dye pollutants [113,133–135]. The techniques for producing magnetic-modified biochar generally fall into two main categories: pre-treatment of raw materials and post-treatment of the produced biochar. In magnetic modification, magnetic iron oxide is typically applied to the surface of biochar to sustain magnetic induction and offer active sites for iron oxide to eliminate pollutants [136]. The magnetic properties of biochar facilitate easy separation from solution, thereby enhancing its removal efficiency. Fakhar *et al*. fabricated a magnetic biochar adsorbent using discarded peels from *Pyrus pyrifolia*, impregnated with FeCl_3_ as a precursor [137]. This magnetic biochar, easily separable, proved efficient in removing methylene blue as a model organic dye pollutant. Textural analysis of the modified magnetic biochar using Brunauer–Emmett–Teller measurements revealed its mesoporous nature. Physicochemical features of the Fe-modified biochar indicated that the impregnation of FeCl_3_ significantly influenced the microstructure of the synthesized Fe-modified biochar, resulting in improved adsorption efficiency and increased magnetic separation of the biochar after-treatment processes. In another study, Enionla *et al*. engineered modified iron oxide biochar from date palm for the removal of methylene blue from wastewater [138]. The findings indicated that the modified iron oxide biochar, with an adsorption capacity of 85.1 mg g^−1^, exhibited significant improvement compared to unmodified biochar (60.1 mg g^−1^) and iron oxide (50 mg g^−1^) at an optimal pH of 8. This improvement was attributed to increased surface area, porosity and the deposition of metal ions on its surface for adsorption. The enhanced methylene blue adsorption by the modified biochar can be ascribed to electrostatic attraction, hydrogen bonding, π–π interactions and potential cationic exchange, as evidenced by Fourier transform infrared, energy-dispersive X-ray and X-ray diffraction analyses. The modified magnetic biochar exhibits improved specific surface area, catalytic adsorption capability, optimized pore size distribution, increased pore volume and enhanced ease of separation, resulting in the effective removal of organic dye pollutants [139–141]. This feature allows for the repeated application of magnetic biochar in wastewater treatment. Furthermore, the repeated use of magnetic biochar reduces the required quantity for water cleanup, mitigating the risk of secondary pollution caused by contaminated biochar leaking into water bodies.

### 5.3. Metal-doping-modified biochar

Researchers have been focusing on the metal doping of biochar, as certain metals can utilize oxygen functionalities as sites for introduction, resulting in stable metal centres on the biochar surface. The introduction of metals alters the surface electronegativity, dispersibility and functional groups of biochar. Metal-modified biochar enhances contaminant removal efficiency and is easily separable from contaminated systems [142–144]. The synthesis, characterization and application of metal-doped biochar have been extensively investigated and frequently documented in recent years, given these advantageous properties. A mesoporous biochar infused with selenium was successfully crafted and utilized for the effective adsorption of reactive orange 16 dye. Liu's equilibrium model exhibited optimal fitting, indicating a maximum adsorption capacity of 538 mg g^−1^ for reactive orange 16 dye. Various mechanisms, such as pore filling, π–π interactions and hydrogen bonding, played roles in the adsorption process between Biochar-Se and reactive orange 16 dye molecules. The formation of metal–oxygen bonds by selenium nanoparticles contributed to the enhanced adsorption of reactive orange 16 dye [145]. Carbon dots doped with copper were created through hydrothermal carbonization at 180°C, utilizing ascorbic acid, urea and copper chloride as precursors. Simultaneously, biochar was produced through the pyrolysis of rice husk at 600°C. The resulting metal-modified biochar demonstrated successful application in removing Congo red dye from an aqueous system. At a catalytic dose of 100 mg, the maximum adsorption capacity reached 437.4 mg g^−1^ for the removal of 20 mg l^−1^ of Congo red dye [146]. Metal doping is employed to enhance the adsorption capabilities by altering the surface properties of biochar. In contrast to unmodified biochar, metal-doped porous biochar exhibits a notable increase in surface acid content and a change in surface electronegativity [147]. These modifications contribute to improved adsorption performance by strengthening acid–base interactions and electrostatic interactions between the adsorbent and dye molecules, respectively. The metal-doping modification increases the presence of oxygen-containing functional groups in biochar, which enhances its ability to adsorb organic dye pollutants. However, this enhancement poses a risk of releasing metal ions and potentially causing secondary contamination.

### 5.4. Heteroatom-modified biochar

The incorporation of heteroatoms, such as N, P and B, stands as a significant approach for modifying biochar composites [148,149]. These heteroatoms are introduced either onto the surface of the carbon matrix or covalently embedded uniformly within the carbon structure on the biochar to enhance its performance in specific applications including water remediation from organic dyes [150,151]. This modification technique allows for the adjustment of the volume and surface characteristics of carbon, thereby altering its physical and chemical properties, including thermal stability, surface chemistry and functionality. Consequently, carbon materials undergo adaptable changes, enabling them to effectively adsorb and remove organic pollutants, such as dyes, from aquatic environments [152]. Ekman *et al*. synthesized N-doped porous biochar from spruce bark waste through a straightforward single-step synthesis using H_3_PO_4_ as the chemical activator [153]. The N-doping modification increased microporosity, proving effective for adsorbing reactive orange 16 dye and treating synthetic effluents with dyes. N-doped biochar exhibited significantly enhanced adsorption capacities compared to non-modified biochar. The high porosity of N-doped biochar mainly facilitated pore filling as the main adsorption mechanism, complemented by other mechanisms such as electrostatic interactions, hydrogen bonding, Lewis acid–base interactions and π–π interactions in the removal of organic dye via the synthesized modified biochar. A nitrogen-doped biochar, utilizing a natural byproduct from birch trees and melamine as the nitrogen dopant, was synthesized for the adsorption of acid red 18 dye from water [154]. The N-doped biochar demonstrated a maximum adsorption capacity of 545.2 mg g^−1^, whereas the non-doped biochar exhibited 444.5 mg g^−1^, marking a 22.6% increase. The removal of acid red 18 dye indicates the involvement of interactions such as electrostatic forces, hydrogen bonds, Lewis acid–base interactions and π–π interactions between the adsorbent and the dye. The addition of heteroatoms to biochar enhances its adsorption capacity by increasing both the specific surface area and the quantity of functional groups.

### 5.5. Metal and heteroatom co-doped modified biochar

Metal–heteroatom doping in biochar involves the introduction of specific metal and non-metal elements (heteroatoms) into the biochar structure. The goal is to enhance its effectiveness in removing organic dyes from different solutions by modifying the biochar's surface properties. This process creates novel functional groups, improving the biochar's adsorption capabilities. Metal–heteroatom doping has the potential to influence the chemical reactivity, surface charge and overall adsorption efficiency of biochar. Transition metals like iron, copper, or manganese serve as active sites for organic dye adsorption, introducing extra binding sites on the biochar surface. Simultaneously, heteroatoms like nitrogen (N), sulfur (S), or phosphorus (P) contribute to altering the biochar's electronic structure, facilitating the formation of new functional groups [155,156]. There is an ongoing commitment to developing biochar with enhanced effectiveness in the removal of organic pollutants and environmental remediation. The incorporation of metals and heteroatoms, such as N, S, or P, stands out as an efficient technique for enhancing the functionality of biochar [157]. The introduction of heteroatoms onto metal-doped biochar appears to be a promising approach to bolster the capacity to remove contaminants and improve the stability and reusability of metal-modified biochar. Notably, nitrogen-doped magnetic biochar demonstrated superior catalytic performance in eliminating organic pollutants compared to both iron–biochar and nitrogen–biochar [158].

## 6. Mechanism of organic dye removal by modified biochar-based adsorption

**Figure 5.**
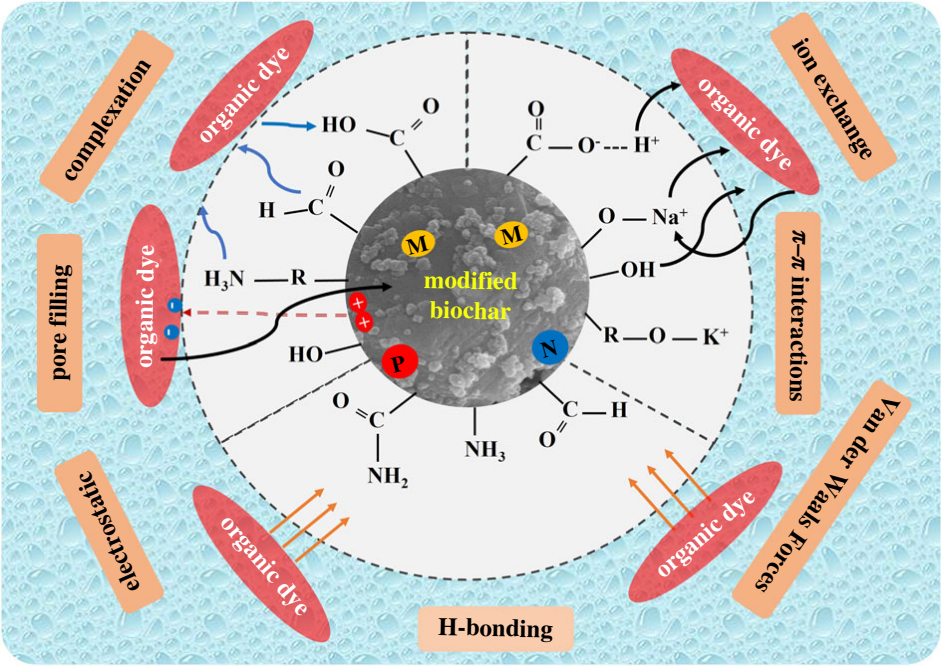
Mechanism of modified biochar adsorption for organic dye removal from aqueous solutions.

The composition of biochar is predominantly influenced by factors such as the raw materials used, pyrolysis temperature, kinetic parameters, coexisting ions and particle size. Various factors contribute to the specific adsorption mechanisms for organic dyes in wastewater, and these mechanisms can vary among different biochar materials [159]. Variations in the chemical composition of organic dye and the presence of distinct surface-active and functional groups among different types of biochar result in diverse adsorption mechanisms for organic dye pollutants. Consequently, comprehending these adsorption mechanisms is crucial as they significantly impact the factors influencing the adsorption process. The adsorption of dye onto biochar surfaces is mainly driven by physical interactions like pore filling, π stacking, van der Waals forces, electrostatic and hydrophobic interactions and hydrogen bonding [130]. Nevertheless, the surface area, porosity and aromaticity of biochar play a crucial role in the physical adsorption process. In modified biochar, increased surface area and pore volume improve contaminant diffusion [160]. Electrostatic interactions manifest between the charged functional groups on dye molecules and the charged sites on the biochar surface [161,162]. Electrostatic interaction involves ionizable functional groups on the adsorbent surface interacting electrostatically with those on the organic dye adsorbate molecules, leading to attraction or repulsion depending on pH levels [163]. pH significantly influences the surface charge of the adsorbent and the extent of dissociation of organic dye functional groups. At pH values greater than the p*K*_a_, organic dye molecules bear a negative charge, while at pH values lower than the p*K*_a_, they remain neutral [164]. Hydrophobic interaction presents another probable mechanism for adsorption, where non-polar groups tend to aggregate in an aqueous medium, thereby minimizing their exposure to water molecules [165]. Hydrogen bonding occurs between the polar groups on the dye molecules and the polar groups on the biochar surface. π–π interactions occur between aromatic rings on the dye molecules and aromatic rings on the biochar surface [161,166]. The extent of aromaticity within an organic dye molecule significantly influences the nature of interactions dictating the mechanism of biochar-based adsorption for dye pollutant removal. Qiu *et al*. [167] suggested that due to their largely planar structures and high degrees of aromaticity, reactive brilliant blue and rhodamine B primarily engage in π–π dispersive interactions with the graphene layers of modified biochar, resulting in their high adsorption capacities. Surface functional groups such as carboxyl and lactones in modified biochar are believed to attenuate these π–π interactions by withdrawing electrons, thereby reducing the electron density on the biochar surface. Conversely, phenolic hydroxyls, possessing electron-donating properties, are anticipated to enhance π–π interactions. Zhang *et al*. explored the use of biochar derived from KOH-modified phenol–formaldehyde resin wood for removing methylene blue and Congo red dyes from aqueous solutions. Their findings revealed that the primary mechanisms responsible for dye removal were pore filling, electrostatic attraction, hydrogen bonding and π–π interactions [127]. In a separate investigation conducted by Wang *et al*., the elimination of rhodamine B from river water was examined utilizing unmodified biochar derived from inexpensive waste cartons. The findings indicated that the adsorption mechanism was primarily governed by physisorption involving van der Waals forces and π–π interactions [168]. A single-step synthesis of inorganically modified meso-biochar led to a highly effective and selective adsorbent for diverse organic dyes. Notably, the iron-modified biochar displayed enhanced adsorption capacity and selectivity for organic pigments. This research revealed that the increased adsorption affinity stemmed from hydrogen bonding and n–π interactions. Furthermore, the molecular structures of the targeted pigments played a key role in the observed enhanced and selective adsorption [162]. The aromatic composition of biochar holds the potential for establishing π-stacking and hydrogen bond interactions with organic dye pollutants. Post-modification of biochar can enhance pore filling, π–π interactions and hydrogen bonding between biochar and dye [169,170]. Hydroxyl and amine groups provide an advantage for π–π interactions, particularly due to the presence of electron-deficient functional groups on the surface of cationic dyes [171]. Figure 5 illustrates the common adsorption mechanisms of modified biochar in the removal of organic dyes from aqueous systems. The mechanism of the adsorption process of organic dyes by modified biochar is influenced significantly by various factors, including the properties of the organic dye adsorbate (e.g. molecular size, polarity, solubility, p*K*_a_, electron distribution), as well as the characteristics of the modified biochar adsorbent, such as specific surface area, surface functional groups and pore size distribution. Additionally, experimental conditions of water like pH, temperature, initial dye concentration, ionic strength and biochar dosage play crucial roles.

## 7. Adsorption factors and biochar regeneration

The adsorption performance is greatly influenced by experimental parameters such as solution pH, temperature, dye pollutant concentration, adsorbent amount, and the presence of co-existing pollutants and water constituents. The adsorption behaviour of organic dye pollutants onto the biochar surface can be comprehensively analysed using adsorption isotherms, kinetics and thermodynamics. Additionally, the type of biochar, including its modification, surface area and porosity, significantly affects adsorption efficiency. These factors also play a crucial role in the regeneration and recyclability of biochar or its modified composites in practical applications. This section offers insights into the factors impacting the adsorption process and the regeneration of modified biochar.

### 7.1. Factors affecting the adsorption process

Biochar finds extensive use in effectively removing organic dyes through adsorption due to its superior performance. In addressing the adsorption of organic dyes in wastewater, it is crucial to thoroughly comprehend the potential environmental issues post-adsorption. This involves a consideration of the economic efficiency of using modified biochar materials for wastewater adsorption, along with aspects like biochar recycling and the management of waste biochar. The adsorption of organic dyes onto biochar and modified biochar is influenced by various factors. One such factor is the pH of the solution, which can impact the ionization of dye molecules and the biochar surface [172,173]. The optimal pH for effective adsorption commonly falls within the range of 5 to 7 [174]. The solution temperature can impact both the solubility of dye molecules and their diffusion onto the biochar surface. As a general trend, the adsorption capacity of both biochar and modified biochar tends to increase with selecting optimum temperature depending on the type of organic dye and the nature of the biochar [46,175]. The concentration of dye in the solution influences the availability of adsorption sites on the biochar surface [176]. The adsorption capacity of both biochar and modified biochar generally rises with increasing dye concentration until the adsorption sites reach saturation. The type of biochar is a key factor affecting adsorption capacity, with biochar featuring high surface area and porosity typically demonstrating greater effectiveness [177,178]. Additionally, modifications to biochar can enhance its adsorption capacity; for instance, the introduction of metal ions can increase the number of available adsorption sites [177,179].

### 7.2. Regeneration of modified biochar

Regenerating and reusing adsorbents in the removal of organic dyes through adsorption pose challenges for researchers. When organic dyes cover the active adsorption and oxidation sites of biochar or modified biochar, the reactivity of the adsorbent diminishes over time. To re-establish its effectiveness, specific treatment processes become essential. Biochar stands out for its recognized reusability through straightforward regeneration, making it a more sustainable choice compared to other materials. The regeneration of biochar-based sorbents is essentially the reverse of the adsorption process [180]. An effective biochar adsorbent should possess reusability and recyclability for practical applications, thereby substantially lowering the cost of biochar sorbents through a repetitive sorption–desorption cycle. The adsorption efficiency of biochar may decline with successive use. Regenerating biochar is a process opposite to adsorption, involving two principles: desorption of adsorbate and decomposition of adsorbate [181]. An effective competitive adsorbent should demonstrate excellent reusability and recycling capabilities for industrial applications, thereby significantly reducing the cost of biochar sorbents through repeated sorption–desorption cycles. There are four primary techniques for regenerating biochar: thermal regeneration [181], solvent reactivation [182], microwave regeneration [183] and supercritical fluid reactivation [182]. Regenerating a biochar adsorbent is influenced by factors such as biochar properties, applied quantity, organic dye type and the chosen regeneration method. Despite these challenges, investigating the reuse and regeneration of biochar for organic dye removal through adsorption is a promising research direction, offering the potential to enhance biochar's sustainability and cost-effectiveness. Several solvents have been employed to regenerate biochar after its application in water and wastewater treatment. Table 4 displays some of these common solvents used for biochar regeneration and activation, along with their efficacy.

**Table 4.** Various solvents used in regenerating biochar for water remediation applications.

biochar	solvents	pollutants	cycles	ref.
dyeing sludge biochar	Fenton reaction (Fe^2+^/H_2_O_2_)	antibiotics	5	[184]
municipal sludge biochar	ethanol assisted ultrasound	tetracycline and ciprofloxacin	6	[185]
Zn-modified biochar	ethanol and distilled water	methylene blue	5	[186]
Zn terephthalate-MOF/Ag biochar	ethanol and acetic acid	Congo red	6	[187]
Co_2_MnO_4_-Ni-modified biochar	methanol	methylene blue, malachite green, and crystal violet	6	[188]
S-doped chitosan/biochar	NaOH	malachite green and rhodamine B	5	[189]
ZnCl_2_-leaves biochar	NaOH	crystal violet	5	[190]
ZnO-modified biochar	HCl	Congo red	5	[110]

### 7.3. Adsorption isotherms, kinetics and thermodynamics

Adsorption isotherm models are important tools for comprehending and predicting how adsorbate molecules interact with surfaces, especially in environmental remediation. These models help define adsorption capacity, surface variation and energy distribution, offering valuable insights into processes such as pollutant removal in wastewater treatment [191]. Different isotherm models, such as Langmuir, Freundlich, Elovich, Jovanovic, Temkin, Halsey, Toth, Kiselev, Sips and others, are employed to assess the adsorption behaviour of organic dye pollutants onto biochar surfaces [192,193]. However, the Langmuir (equation (7.1)) and Freundlich (equation (7.2)) models are frequently preferred for describing this phenomenon:

7.1 $$q_{e}=\frac{C_{e}q_{m}K_{L}}{1+K_{L}C_{e}}$$

and

7.2 $$q_{e}=K_{f}C_{e}^{1/n}.$$

Here, *q*_e_ represents the equilibrium adsorption, *C*_e_ stands for the equilibrium concentration, *K*_L_ is the Langmuir constant, *q*_m_ denotes the maximum or optimal adsorption capacity, *K*_f_ signifies the Freundlich constant and *n* represents the Freundlich constant indicating the extent of adsorption on the surface of the biochar adsorbent.

Several kinetic models, including pseudo-first-order and pseudo-second-order models, as well as Elovich, intra-particle, Bangham and film diffusion models, have been formulated to explain dynamic behaviour, comprehend the interaction between adsorbent and adsorbate, and gain insights into the adsorption mechanism of organic dyes by modified biochar [194]. Among these, the pseudo-first-order and pseudo-second-order models are most frequently employed. In chemical kinetics, the prefix ‘pseudo’ signifies that at least one influencing factor remains constant and is not factored into the mathematical expression. This poses a challenge in adsorption kinetics because changes in liquid phase concentration are necessary to confirm adsorption. Therefore, the prefix ‘pseudo’ is applied to these equations as they focus on the adsorbate concentration in the solid phase rather than in the liquid phase [195].

To gain deeper insights into processes like adsorption kinetics, researchers examine various thermodynamic parameters such as changes in Gibbs free energy (ΔG°), entropy (ΔS°) and enthalpy (ΔH°) [196]. A negative ΔG° indicates the spontaneity of the adsorption process, while a positive ΔS° reflects increased randomness of adsorbate molecules/ions on the adsorbent surface compared to the solution. ΔH° is utilized to discern whether adsorption is exothermic (negative ΔH°) or endothermic (positive ΔH°) in nature [197]. The adsorption process, whether exothermic or endothermic, is connected to the physicochemical attributes of organic dye pollutants, such as their molecule size and the presence of functional groups. Similarly, the physicochemical properties of the biochar, like variations in functional groups between pristine and modified forms, also play a role in this process.

## 8. Modified biochar adsorbent: a promising solution with challenges and opportunities

Biochar and its modified composites are efficiently used in wastewater treatment, especially for removing organic dye pollutants from water, as eco-friendly adsorbents. Biochar distinguishes itself from other adsorbents due to its cost-effectiveness, broad applicability, environmental benefits and high adsorption capacity [115]. Various carbonaceous organic sources, including agricultural waste and residues, municipal and industrial byproducts and activated sludge, serve as biochar feedstock [103,114]. The adsorption characteristics vary among materials derived from different raw sources. Choosing economical and eco-friendly raw materials is vital for preparing efficient adsorbents. Biochar is commonly employed for the removal of organic dye pollutants from wastewater due to its extensive surface area, high porosity, oxygen-containing functional groups, low preparation cost and ease of modification [145]. Limited research has focused on the production costs of biochar-based adsorbents. Despite existing cost-effective production methods and the potential for multiple reuse cycles, further exploration is crucial to confirm the efficiency and affordability of biochar, especially for pilot-scale implementation in diverse applications. Modified biochar exhibits potential as an adsorbent for eliminating harmful organic dye pollutants [153]. Ongoing research on the modification and application of modified biochar is progressing, with a significant number of articles published annually. Table 5 provides a summary of recent articles detailing the effective removal of various organic dyes using modified biochar.

**Table 5.** Adsorption capacities of various biochar and modified biochar for water remediation from organic dyes.

feedstock	modifier	organic dye	capacity (mg g^−1^)	ref.
phenol–formaldehyde resin modified wood	KOH	methylene blue	1112.32	[127]
		Congo red	3472.22	
waste carton	biochar	rhodamine B	222.6	[168]
*Retinervus luffae fructus*	ZnCl_2_	basic red 46	181.5	[198]
poultry manure	Zn-terephthalate-MOF	Congo red	416.6	[187]
sorghum straw	Fe_3_O_4_	methyl blue	153.4	[162]
Douglas fir	Fe_3_O_4_	bromophenol blue	448.0	[199]
sawdust	H_2_SO_4_, O_3_, triethylenetetramine	methyl blue	568	[200]
cassava residues	NH_4_Cl	food red 17	131	[201]
areca nut shell	CeO_2_	methylene blue	492.2	[202]
sawdust	ZnCl_2_	malachite green	318.47	[203]
*Blighia* *sapida* wastes	HCl	allura red	15.17	[204]

Biochar-based adsorption is a promising technique for removing organic dyes from wastewater, but it faces several challenges that hinder its widespread adoption. These challenges include the generation of secondary pollutants, high regeneration and separation costs, pH sensitivity and potential scaling-up difficulties. Moreover, some organic dyes are resistant to adsorption by unmodified biochar, and the adsorption efficiency is also influenced by wastewater pH, making process optimization challenging for different wastewater streams. Furthermore, unmodified biochar generally has a low surface area and lacks functional groups, which are crucial for effective adsorption. Developing modified biochar adsorbents in an eco-friendly and efficient manner is an area of active research. Current biochar preparation methods often rely on harmful chemicals and suffer from low biochar yields, resulting in wasted precursor materials and increased costs. The type of biomass used also influences biochar production. Utilizing contaminated feedstock in co-pyrolysis, like sewage sludge or plant biomass, might release harmful pollutants. Using plastics to improve the process can raise new concerns, as microplastics are increasingly found in the environment. More research is urgently needed to understand the safety risks and set safe operating limits for this technology. Currently, most studies conducted on biochar adsorption of organic dye pollutants in water concentrate on single-dye adsorption. However, real-world and industrial wastewater typically involves a mixture of pollutants, making the adsorption mechanism more complex. Hence, future studies should explore the competitive adsorption of modified biochar materials for various organic pollutants within actual wastewater complex pollutant systems. Nevertheless, modified biochar-based adsorption holds promise as a technique for removing organic dyes from wastewater. Ongoing research efforts are crucial to address the challenges associated with biochar preparation and modification, enabling the development of more efficient and affordable organic dye wastewater treatment using this sustainable adsorbent.

## 9. Conclusion and outlook

Organic dye pollutants present a significant environmental risk due to their toxicity, carcinogenic potential and resistance to conventional removal techniques. Hence, it is crucial to treat them before releasing them into aquatic environments. Modified biochar has emerged as a promising, sustainable and effective method for treating organic dyes in wastewater. This review summarizes the latest developments in using modified biochar as an eco-friendly adsorbent for removing various organic dye pollutants from industrial wastewater. However, practical implementation of modified biochar-based adsorption for organic dye decontamination faces several unresolved challenges. Future research should focus on enhancing efficiency, cost-effectiveness, matrix compatibility, catalyst stability, simplistic regeneration methods and seamless integration with existing treatment systems. Adsorption utilizing biochar provides a promising and efficient means of removing organic dyes from wastewater. The success of this treatment method relies on the inherent properties of the biochar and the implemented modification strategies. These strategies encompass augmenting surface area, improving porosity, enhancing magnetic properties and introducing functional chelating groups to facilitate the capture of pollutants. Magnetic biochar, an inventive bio-carbon material, exhibits both advantageous adsorbent properties and magnetic characteristics. Furthermore, the cost associated with activation and regeneration techniques plays a pivotal role in ensuring the reusability and stability of the biochar. Despite biocarbon materials, including biochar and its modified composites, being predominantly used as activator catalysts in advanced oxidation techniques for pollutant treatment, the effectiveness of biochar adsorption for the complete removal of organic dyes is still a subject of consideration. Proper disposal of biochar after use is essential for ensuring the sustainability and environmental impact of biochar-based adsorption in wastewater treatment. The stability and reusability of modified biochar are key factors for practical application. Disposal methods may involve incorporating biochar into the soil as a soil amendment, using it for energy recovery or composting. Additionally, exploring options for recycling or reusing spent biochar could help address disposal concerns. While many studies have shown the effectiveness of modified biochar in laboratory settings, there is a lack of commercial-scale and field studies. The future use of modified biochar for removing organic dyes from contaminated water depends on the development of large-scale, long-term field investigations. Moreover, further research is needed to confirm the scalability of biochar as an environmentally friendly adsorbent on an industrial scale, including the exploration of biochar regeneration and waste management techniques.

## Data Availability

This article has no additional data.
